# Macrophage activation and invasion by *P. gingivalis* is modulated by PPAD and accessory fimbriae subunits

**DOI:** 10.1080/20002297.2026.2638646

**Published:** 2026-03-03

**Authors:** Aleksandra Wielento, Dominika M. Drapała, Marina Terekhova, Anna Jacuła, Maxim N. Artyomov, Jakub Kochan, Yoshiaki Hasegawa, Danuta Mizgalska, Juhi Bagaitkar, Aleksander M. Grabiec, Jan Potempa

**Affiliations:** a Department of Microbiology, Faculty of Biochemistry, Biophysics and Biotechnology, Jagiellonian University, Krakow, Poland; b Current affiliation: Centre for Translational Microbiome Research, Department of Microbiology, Tumor and Cell Biology, Karolinska Institute, Stockholm, Sweden; c Doctoral School of Exact and Natural Sciences, Jagiellonian University, Kraków, Poland; d Department of Pathology and Immunology, Division of Immunobiology, Washington University School of Medicine, St. Louis, MO, United States of America; e Department of Cell Biochemistry, Faculty of Biochemistry, Biophysics and Biotechnology, Jagiellonian University, Krakow, Poland; f Department of Microbiology, School of Dentistry, Aichi Gakuin University, Nagoya, Japan; g Center for Microbial Pathogenesis, Abigail Wexner Research Institute at Nationwide Children's Hospital, Columbus, OH, United States of America; h Department of Oral Immunology and Infectious Diseases, School of Dentistry, University of Louisville, Louisville, KY, United States of America

**Keywords:** *Porphyromonas gingivalis*, macrophages, Toll-like receptor 2, PPAD, fimbriae, accessory fimbriae subunits, immune evasion

## Abstract

**Background:**

*Porphyromonas gingivalis* is a master manipulator of host immune responses in the periodontium. Peptidyl arginine deiminase (PPAD), a recently identified virulence factor of *P*. *gingivalis*, responsible for citrullination of both host- and bacterium-derived proteins and peptides, also plays a key role in hijacking immune responses. While PPAD's modification of fimbrial subunits (FimCDE) affects TLR2 signalling in fibroblasts, its effects on immune cells remain unclear.

**Methods:**

Human monocyte-derived macrophages (MDMs) were stimulated with wild-type or mutant *P. gingivalis* strains and isolated fimbriae. Inflammatory responses were assessed by measuring cytokine expression and secretion, transcriptional changes using RNA-seq and Pi3K/Akt pathway activation, as well as bacterial invasion through flow cytometry, fluorescent microscopy, and intracellular survival assays.

**Results:**

PPAD-modified fimbriae stimulated MDM pro-inflammatory responses, such as PGE2-related gene expression and cytokine secretion, while fimbriae from accessory subunit mutants failed to induce inflammation. PPAD modification of accessory fimbrial subunits was found to protect *P. gingivalis* from macrophage killing. PPAD and accessory fimbriae subunits participated in complex immune evasion strategies, upregulating genes linked to viral infections or T cell interactions.

**Conclusions:**

These findings highlight the importance of protein citrullination in TLR2-related signaling and offer insight into how *P. gingivalis* evades host immune responses.

## Key messages

- PPAD-modified fimbriae enhanced expression of enzymes in the PGE2 synthesis pathway and secretion of cytokines interleukin (IL)-6 and IL-8 from human monocyte-derived macrophage (MDM).
- PPAD-modified accessory fimbrial subunits protected *P. gingivalis* from killing by macrophages.
- PPAD and accessory fimbriae subunits are important in complex immune evasion strategies, upregulating genes associated with viral infections or interactions with T cells.

## Introduction

Periodontitis is a chronic inflammatory disease that results in the destruction of the hard and soft tissues supporting the teeth and poses a risk for systemic and degenerative diseases [1]. The primary cause of chronic inflammation in periodontitis is the dysbiotic oral microbiome, with *Porphyromonas gingivalis* considered one of the key pathogens [2]. *P. gingivalis* produces a variety of virulence factors, such as lipopolysaccharide (LPS), polysaccharide capsule, gingipains and other proteases, fimbriae, and peptidyl arginine deiminase (PPAD) [3], which are exposed on the bacterial surface or released into the environment in soluble forms and/or linked to outer membrane vesicles (OMVs) [4]. All of these contribute to *P. gingivalis* resistance to killing by the host immune system. Among them, PPAD is a unique virulence factor in the prokaryote kingdom because it is encoded and expressed only by *P. gingivalis* and *Porphyromonas gulae*, a closely related species in cats and dogs [5]. Its primary role is to convert C-terminal Arg residues into citrulline in proteins and peptides derived from bacteria and the host [6].

Toll-like receptor-2 (TLR2) plays a key role in recognising *P. gingivalis* by host cells [7]. TLR2 is found on various immune cells, including macrophages, dendritic cells, and neutrophils, as well as on epithelial cells and fibroblasts in the oral cavity [8]. *P. gingivalis* fimbriae are among the most important TLR2 ligands in periodontitis [9], involved in bacterial adhesion to surfaces, host cells, and other bacterial species during biofilm formation [10]. PPAD-dependent modification of fimbriae has been shown to boost TLR2 activation, while the absence of PPAD reduces the pro-inflammatory responses triggered by *P. gingivalis* fimbriae in primary human gingival fibroblasts (PHGFs) [11].

The fimbriae shaft is composed of a FimA polymer associated with accessory fimbriae subunits (FimC, FimD, and FimE), with the FimA filament anchored in the outer membrane by the lipoprotein FimB [10]. PPAD-modified accessory fimbrial subunits, whether alone or in combination, but not FimA, were found to be required for TLR2 activation and for triggering pro-inflammatory responses in PHGFs [12]. To date, however, nothing is known about the role of bacterial protein citrullination, particularly PPAD-dependent modification of fimbriae, in immune cell responses [13,14].

Macrophages are vital immune cells that eliminate bacteria, present antigens to T cells, coordinate inflammatory responses through cytokine secretion, and help maintain tissue homoeostasis [15]. In periodontitis, *P. gingivalis* influences macrophage polarisation, promoting the M1 (pro-inflammatory) phenotype, as TLR2/4 activation increases cytokine release and bone resorption [16]. Conversely, *P. gingivalis* also inhibits nitric oxide (NO) production, reducing the phagocytic ability of macrophages [17]. Therefore, macrophages contribute to creating favourable conditions for *P. gingivalis* growth during periodontitis development.

Our findings emphasise the role of bacterial protein citrullination in TLR2-related signalling and offer insights into how *P. gingivalis* hijacks host pro-inflammatory responses to enhance its survival and growth *in vivo*.

## Methods

### Bacterial culture and cell infections

Bacterial culture and bacteria preparation for cell infections were performed as detailed in ref. [11]. To clarify the role of fimbriae and their citrullination in *P. gingivalis* interaction with macrophages, we used the fibrillated ATCC 33277 strain and mutants in the ATCC 33277 genetic background, which are deficient in PPAD (∆PPAD) [18], FimA (∆FimA) [19], FimC (OZ5001C, ∆FimC) [20], and FimE (K04, ∆FimE) [21], as well as the mutant strain expressing catalytically inactive PPAD (C351A PPAD) [18]. Additionally, to confirm our findings, we used the *P. gingivalis* W83 wild-type strain, which lacks major fimbriae (FimA) due to mutations in the regulatory gene (*fimS*) within the fimSR operon [20]. All tested mutant strains were cultured on plates supplemented with erythromycin (5 µg/mL), except for PPAD^C351A^, which was cultured on plates containing tetracycline (1 µg/mL). The timing of infection and the multiplicity of infection (MOI) are indicated in each figure legend.

### Isolation of fimbriae, quality assessment, and cell treatment with fimbriae

Fimbriae were isolated from bacterial wash fluid as described earlier [11]. SDS-PAGE was performed to verify purity, and a western blot was used for the final detection of the protein of interest [11,12]. The concentration of fimbriae used and the duration of stimulation are indicated in each figure legend.

### Monocyte isolation and macrophage culture

Human blood collected from de-identified healthy individuals was purchased from the Regional Blood Donation and Treatment Centre in Krakow. The detailed protocol for isolating monocytes (CD14^+^ cells) from peripheral blood was described previously [22]. Briefly, peripheral blood mononuclear cells (PBMCs) were isolated from human blood using density gradient centrifugation. Monocytes were separated from the PBMC fraction using magnetic sorting beads (Miltenyi Biotec). Monocytes were then differentiated into monocyte-derived macrophages (MDMs) by culturing them for 1 week in RPMI 1640 with 10% FBS and 50 ng/mL human recombinant GM-CSF (Biolegend). Before infection, the culture medium was replaced with RPMI 1640 containing 2% FBS, and for flow cytometry, the medium was replaced with RPMI 1640 without serum. For widefield microscopy, the medium was replaced with RPMI 1640 without serum.

#### Limulus amebocyte lysate (LAL) test and endotoxin removal

The amount of LPS in fimbriae preparations was measured using commercially available kits (Pierce LAL Chromogenic Endotoxin Quantitation Kit, Thermo Fisher). The removal of residual LPS from fimbriae preps was done using a commercial endotoxin removal resin (Pierce High Capacity Endotoxin Removal Spin Columns, Thermo Fisher) following the manufacturer's protocol.

#### TLR4 blocking and AKT phosphorylation inhibition

Cells were treated for 30 min with a TLR4 neutralising antibody or mouse IgG1 isotype control (both from Invivogen) at 5 µg/mL before treatment with fimbriae. To inhibit phosphorylation of AKT, cells were pretreated with 10 µM Akt inhibitor VIII (Cayman Chemical) for 30 min prior to infection.

#### RNA isolation and quantitative (q)PCR

Reference [11] provides a full description of the procedure, including the list of primer sequences used for qPCR.

#### ELISA

Concentration of IL-6 and IL-8 in cell supernatants was assessed using commercially available kits ELISA MAX (Biolegend) according to the manufacturer's instructions.

#### Western-blot

Cell lysates prepared in Laemmli's buffer containing 2% SDS, 10% glycerol, and 125 mM Tris-HCl, pH 6.8, were standardised for total protein concentration and subjected to SDS-PAGE electrophoresis. Gels were prepared using the TGX Fast Cast Acrylamide kit (Bio-Rad). Proteins were transferred using the Trans-Blot Turbo system (Bio-Rad). A specific band corresponding to the protein of interest was detected by primary antibodies against *p*-Akt (#4060), Akt (#9272S), and *β*-actin (#4967) (all from Cell Signalling Technology) and secondary anti-rabbit antibodies conjugated with HRP (Dako). Blots were developed using the Clarity Western ECL Substrate (Bio-Rad) and visualised with the ChemiDoc MP Imaging System and the ImageLab software (Bio-Rad).

#### Intracellular survival assay

Cells were infected with various strains of *P. gingivalis* for 30 min, then the non-internalised bacteria were removed by washing with PBS, and the medium was replaced with fresh RPMI containing 2% FBS with or without antibiotics: gentamicin (0.2 mg/ml) and metronidazole (0.3 mg/ml). After 1 h of incubation, the cells were washed again, and the medium was replaced once more with fresh RPMI 2% FBS without antibiotics for 1 h. Next, the cells were washed, lysed in sterile distilled water for approximately 20 min, and serial dilutions of the lysates were plated on BHI blood agar plates. The plates were incubated for 5 days at 37 °C in anaerobic conditions, after which bacterial colonies were counted.

#### Bacteria staining

Bacterial suspensions prepared for infections as described in [13]. were adjusted to OD_600_ = 3 (equivalent to 3 × 10^9^ bacteria/mL) in PBS (for CFSE staining) or 100 mM sodium bicarbonate, pH 8.5 (for pHRODO staining). Then, 5 µM CFSE (Carboxyfluorescein succinimidyl ester, CellTrace CFSE Cell Proliferation Kit, Life Sciences) or 5 µM pHRODO (pHrodo-Red succinimidyl ester, Life Sciences) were added to the bacteria. The bacteria were incubated for 30 min at 37 °C in the dark. Labelled bacteria were washed twice with PBS to remove any unbound dye and resuspended at OD_600_ = 1 in PBS.

#### Flow cytometry

After infection with labelled bacteria, macrophages were rinsed with PBS at room temperature and then collected using ice-cold PBS to stop phagocytosis and adhesion, as well as to detach cells from the culture plate. Cells were analysed by flow cytometry with an LSRFortessa (BD Biosciences). Phagocytosis and adhesion are expressed as the percentage of CFSE- or pHRODO-positive cells. Data were analysed using FlowJo software.

#### Widefield fluorescence microscopy

Isolated CD14^+^ cells were seeded on glass coverslips and allowed to differentiate into macrophages for 1 week. Cells were infected with CFSE-labelled *P. gingivalis* strains for 30 min. All staining steps were performed at room temperature in the dark. After infection, cells were washed once with PBS and fixed with 4% formaldehyde for 15 min. Cells were then washed three times with PBS and permeabilized using permeabilization buffer (0.5% Triton X-100, 5% FBS in PBS) for 45 min. Next, actin filaments were stained with Alexa Fluor 647 phalloidin (Invitrogen) at 16.5 nM in permeabilization buffer for 1 h. After three washes with PBS, nuclei were stained with DAPI at 1 µg/ml in PBS for 15 min and rinsed once with PBS. Coverslips with fixed and stained cells were mounted onto glass microscopy slides using ProLong Glass Antifade Mountant (Invitrogen) and left overnight. Imaging was performed using an oil-immersion 100× objective and an IX83 inverted widefield fluorescence microscope (Olympus). Images were captured as single z-planes. Fiji processing software was used to generate the images.

#### RNA-Seq analysis of infected hMDMs

The RNeasy kit (Qiagen) was used to isolate total RNA from MDMs. Paired-end reads from bulk RNA-seq were aligned with STAR v2.7.0f [23]. Gene counts were obtained from the number of uniquely aligned, unambiguous reads using featureCount v2.0.0 [24]. Alignment and gene counts were generated against the GRCh38 (GENCODE release 44) genome assembly. RNA alignment metrics were assessed with the CollectRnaSeqMetrics function from Picard v2.21.1. Batch effects from individual donors were removed using the ComBat-seq function from the sva package v3.38.0 [25]. The DESeq2 pipeline v1.30.1 [26] was used to normalise batch-corrected counts and perform differential expression analysis. Genes with fewer than 10 counts were filtered out. Principal component analysis (PCA) plots were created with the plotPCA function from DESeq2 v1.30.1 [26] after variance stabilising transformation. Gene set enrichment analysis was performed on a pre-ranked list based on Wald statistics for DESeq2 [26] outputs, using the R package fgsea v1.25.2 [27]. Pathway enrichment was conducted using the canonical pathway database collection accessed through the msigdb R package v2.1 [28]. Bar and volcano plots were generated with the ggplot2 package v3.3.2 [29]. Heatmaps were created using the heatmap function from the relevant package v1.0.12 [30].

#### Statistical analyses

Data are presented as mean ± SEM unless otherwise indicated. All experiments were conducted in at least three independent experiments using cells isolated from different donors. One-way analysis of variance (ANOVA) followed by the Dunnett multiple comparison test was used for analysing the data unless otherwise indicated. A *p*-value of <0.05 was considered statistically significant. Statistical analysis was performed with GraphPad Prism 8.02 (GraphPad Software, Inc.).

## Results

### PPAD-dependent modification of fimbriae contributes to pro-inflammatory activation of hMDMs and is triggered by accessory fimbriae subunits

**Figure 1.**
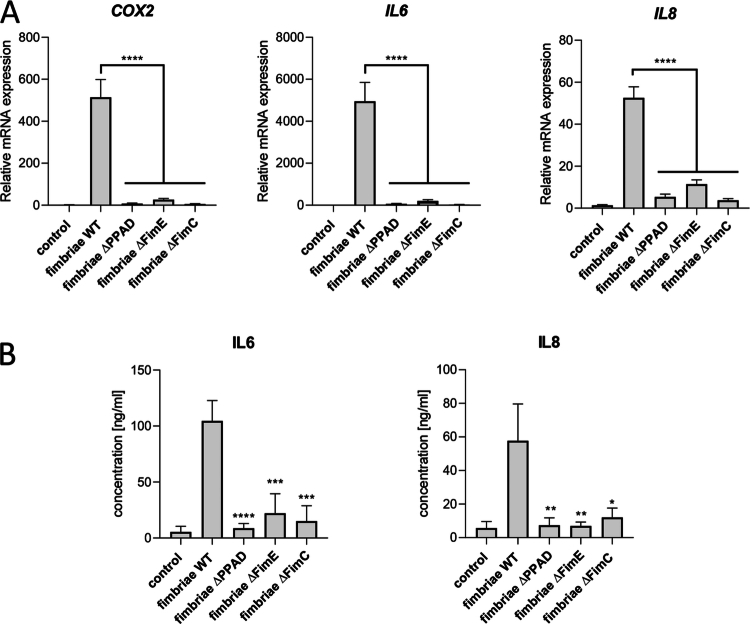
*P. gingivalis* strains with fimbriae depleted of all accessory subunits (DAP) poorly activate MDM pro-inflammatory responses. MDMs were treated for A) 4 h or B) 24 h with purified fimbriae (1 μg/ml) from various *P. gingivalis* strains, including ATCC 33277 WT (fimbriae WT) and ∆PPAD mutant (fimbriae ∆PPAD), as well as mutants of accessory fimbrial subunits, ∆*fimE* mutant (fimbriae ∆FimE) and ∆*fimC* mutant (fimbriae ∆FimC). A) Relative mRNA expression of PGE2 synthesis-related gene *COX2* and cytokines *IL6* and *IL8*; *n* = 4–7. B) Secretion of IL-6 and IL-8; *n* = 5–9. Data are presented as the mean ± SEM compared to WT fimbriae, with significance levels indicated as ****p < 0.0001; ***p < 0.001; **p < 0.01; *p < 0.05.

Based on our previous research focusing on reporter cells or gingival fibroblasts [11,12], we hypothesised that citrullinated accessory fimbriae subunits are also essential for macrophage immune responses to *P. gingivalis*. To test this hypothesis, we treated hMDMs, our study's model, with purified fimbriae from ATCC 33277 wild-type (WT) and its isogenic PPAD-deficient strain (∆PPAD), along with accessory fimbriae subunit mutants (∆*fimC* and ∆*fimE*). Since the assembly of accessory fimbriae subunits on the tip of the FimA shaft depends on the expression of all three subunits, knocking out any accessory *fim* gene results in the absence of the other accessory fimbriae subunits [21]. Compared to fimbriae from the parental strain, fimbriae from the ∆PPAD strain and from strains lacking accessory fimbriae subunits (DAP fimbriae) showed a significantly reduced ability to induce the expression of the PGE2 synthesis pathway gene (*COX2*) and pro-inflammatory cytokines (*IL6* and *IL8*) in hMDMs (Figure 1A), and to stimulate IL-6 and IL-8 secretion (Figure 1B). These results suggest that the accessory fimbriae subunits, potentially citrullinated by PPAD, are weak activators of pro-inflammatory responses in hMDMs.

### The residual LPS in fimbriae preparations has a negligible impact on hMDM response to *P. gingivalis* fimbriae

**Figure 2.**
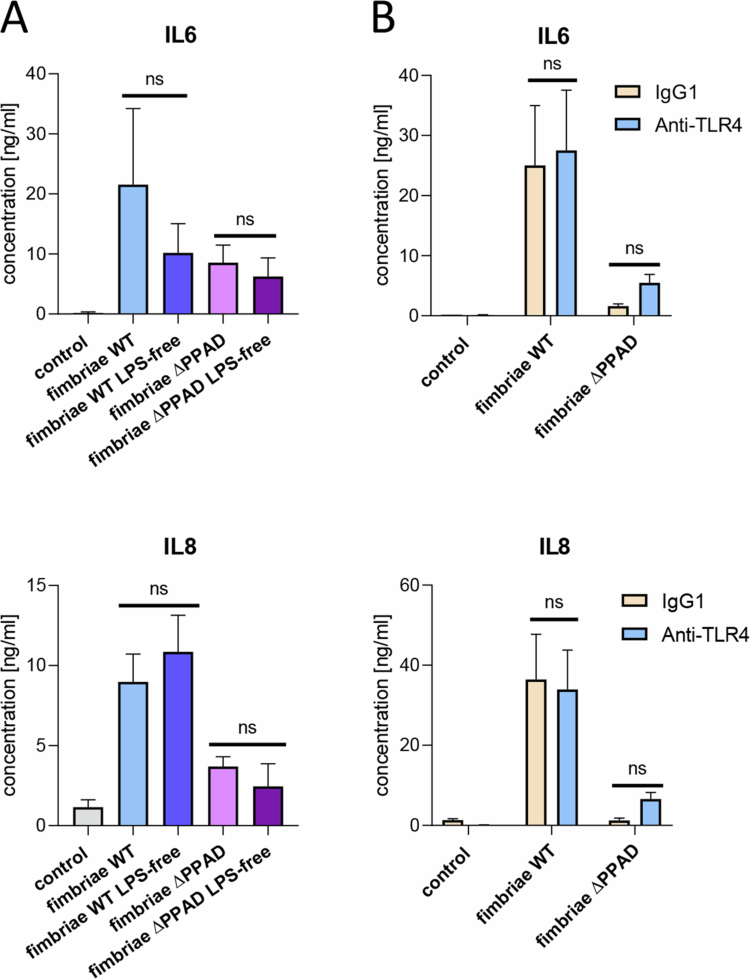
LPS contamination in fimbriae preparations is negligible and does not influence the pro-inflammatory activation of MDMs. A) MDMs were treated for 24 h with purified fimbriae (1 μg/ml) from ATCC 33277 WT (fimbriae WT) and ∆PPAD mutant (fimbriae ∆PPAD) strains, as well as LPS-free fimbriae from both strains; *n* = 3. B) hMDMs were preincubated for 30 min with control (IgG1) or TLR4-blocking antibody (anti-TLR4), then treated for 24 h with purified fimbriae (1 μg/ml) from ATCC 33277 WT (fimbriae WT) and the ∆*ppad* mutant (fimbriae ∆PPAD) strains; *n* = 3. Levels of IL-6 and IL-8 secretion were subsequently measured. Data are presented as mean ± SEM. ‘ns’ indicates not statistically significant. Statistically significant differences are shown in A) between LPS-free fimbriae and fimbriae without the LPS removal step; in B) between IgG1 and anti-TLR4 pretreated samples.

Since purified fimbriae may be contaminated with LPS, residual LPS was removed from fimbriae preparations using an endotoxin removal resin, and the depletion of LPS was confirmed with an endotoxin detection kit. hMDMs were then treated with fimbriae isolated from the WT ATCC 33277 strain and its isogenic ∆PPAD mutant, which were either subjected to LPS removal or not. The levels of secreted IL-6 and IL-8 were measured. Cell responses to fimbriae remained unaffected by LPS removal (Figure 2A), indicating that LPS does not significantly contribute to macrophage stimulation by fimbriae. Notably, a modest, non-significant trend toward higher IL-6 levels in response to non-depleted fimbriae was observed. However, this variation likely reflects donor-dependent biological variability and does not alter the overall conclusion that IL-6 production by fimbriae is largely independent of residual LPS. These results were independently verified by treating hMDMs with a TLR4-neutralising antibody before exposure to fimbriae, which showed that blocking the LPS-TLR4 interaction did not alter the cell responses (Figure 2B). Collectively, these findings demonstrate that any residual LPS in the *P. gingivalis* fimbriae had minimal impact on hMDM inflammatory activation, supporting the notion that, as previously shown, the responses are likely triggered by fimbrial proteins recognised by TLR2 [11].

### PPAD activity and accessory fimbrial subunits influence *P. gingivalis* uptake by hMDMs

**Figure 3.**
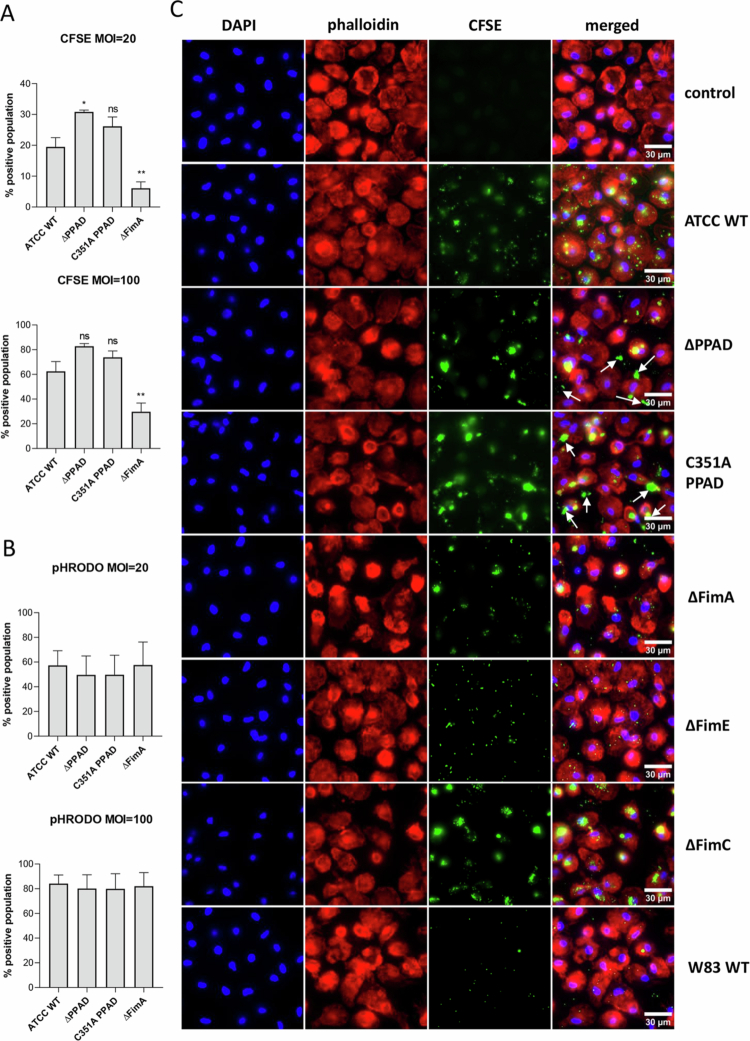
PPAD and fimbriae mutants show differences in adhesion and safe entry to host cells. Flow cytometry analysis of MDMs infected for 30 min at MOI = 20 or 100 with *P. gingivalis* ATCC 33277 WT and PPAD mutant strains—including its isogenic total (∆PPAD) and catalytically inactive (C351A PPAD) strains, as well as fimbriae (∆FimA) mutants—labelled with A) CFSE or B) pHRODO-Red (pHRODO). Phagocytosis and adhesion rates were determined as the percentages of CFSE and pHRODO-Red positive cells, relative to the WT strain; *n* = 4. Results are shown as mean ± SEM. **p < 0.01; *p < 0.05; ns, not significant. Comparisons are made to the ATCC WT strain. C) Widefield fluorescence microscopy of MDMs infected for 30 min at MOI 100 with CFSE-labelled *P. gingivalis* ATCC WT, ∆PPAD, C351A PPAD, ∆FimA, ∆FimE ∆FimC, and W83 strains. Nuclei were stained with DAPI, and actin was stained with phalloidin conjugated to Alexa Fluor 647. Scale bars: 30 µm. Images are representative of three independent experiments. White arrows indicate bacteria that are not internalised.

Next, the mechanism of *P. gingivalis* entry into host cells was examined by determining whether PPAD-modified fimbriae are vital for *P. gingivalis* entry and/or phagocytosis. hMDMs were infected with CFSE-labelled *P. gingivalis* strains: ATCC 33277 WT (ATCC WT), and ATCC 33277-derived strains: ΔPPAD, a catalytically inactive PPAD mutant (C351A), and a fimbriae-deficient mutant (ΔFimA) (Figure 3A). Because the lack of appendages required for attachment to the cell surface [31], the FimA-null mutant was taken up by and/or adhered to hMDMs at significantly lower levels than the ATCC WT strain at both tested MOIs. In contrast, both PPAD mutants exhibited a higher percentage of CFSE-positive cells than the WT strain, indicating enhanced attachment to the cell surface. To differentiate surface-bound from internalised bacteria, we used pHRODO-labelled *P. gingivalis*, which fluoresces in acidic phagolyasosomes [32]. However, no differences in phagocytosis were observed at both tested MOIs (Figure 3B). Overall, these findings suggest that PPAD mutants stay attached to the cell surface, while both adhesion and internalisation of fimbriae mutants by macrophages are impaired.

To gain a more detailed understanding of how *P. gingivalis* strains interact with macrophages, we visualised phagocytosed or adhered CFSE-labelled bacteria using fluorescent microscopy. An MOI of 100 was used to better observe differences between the two *P. gingivalis* strains. The uptake of the ATCC WT and the afimbriated W83 strain by hMDMs differed significantly, with W83 being phagocytosed in low numbers (Figure 3C). The intracellular fluorescence of the major fimbriae-deficient mutant (ΔFimA) was lower than that of the ATCC WT strain but higher than W83. This difference is likely due to the presence of minor fimbriae in the FimA-null strain [33], while W83 is a naturally complete fimbriae mutant [34]. The phagocytosis levels of the accessory fimbriae subunit mutants (ΔFimE and ΔFimC) were similar to those of the ATCC WT (Figure 3C), indicating that the FimA shaft alone is enough to trigger effective uptake of *P. gingivalis* by macrophages. Interestingly, however, the accessory fimbriae subunits seem to contribute to *P. gingivalis*' intracellular survival, including resistance to macrophage killing, and are responsible for inducing inflammatory responses (Suppl. Figure 1) [35]. In contrast to the FimE- and FimC-null mutants, the PPAD mutants (∆PPAD and C351A PPAD) were plentiful on the cell surface and free in the extracellular environment (Figure 3C, white arrows). The finding of large numbers of PPAD mutant cells surrounding macrophages suggested the need to assess bacterial adhesion to glass coverslips used to seed macrophages. Remarkably, significantly more PPAD mutants than the ATCC WT adhered to glass, often in the form of aggregates, in contrast to the W83 strain and all fimbriae mutants, which adhere purely to glass (data not shown). These results suggest that the adhesive properties of ATCC WT *P. gingivalis* depend on FimA and/or accessory subunits and are affected by PPAD, which aligns with findings that adhesion and biofilm formation are higher in strains lacking PPAD compared to ATCC WT [36]. Overall, these findings show that PPAD is vital for the effective uptake of *P. gingivalis* by macrophages and promotes bacterial survival within phagocytes. While the accessory fimbriae subunits are less crucial for host cell invasion, they are key for the long-term survival of *P. gingivalis* inside host cells.

### Citrullinated *P. gingivalis* proteins modulate Akt activation

**Figure 4.**
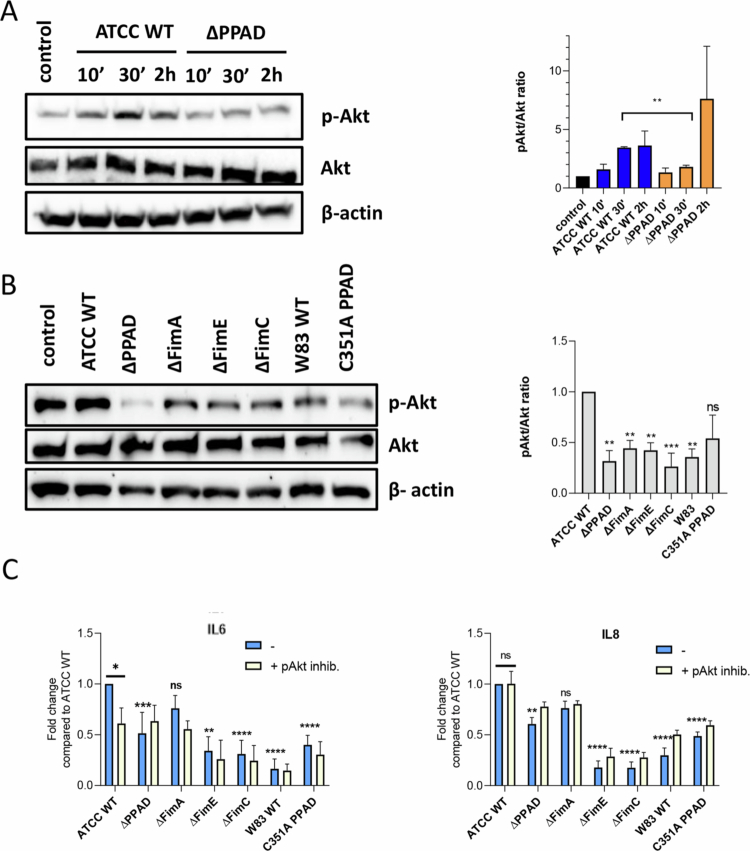
Phosphorylation of Akt is enhanced by PPAD and accessory fimbriae subunits, with the PI3K/Akt pathway being essential for IL-6 but not for IL-8 secretion. MDMs were infected for A) 10 min, 30 min, or 2 h with *P. gingivalis* ATCC 33277 WT strain (ATCC WT) and its isogenic PPAD mutant (∆PPAD), or B) for 30  min with ATCC 33277-derived PPAD: total (∆PPAD) and catalytically inactive (C351A PPAD), fimbriae: main FimA subunit (∆*fimA*), accessory subunits FimE (∆*fimE*), and FimC (∆*fimC*) mutants, or the W83 WT strain at MOI 20. Akt phosphorylation levels were determined by western blot analysis, with total Akt as a control and *β*-actin as the loading control. Representative blots are shown, and densitometry results (*n* = 3) are presented as mean ± SEM. ***p < 0.001; **p < 0.01; *p < 0.05; ns, not significant. In B), data are compared with the ATCC WT strain. C) MDMs were pretreated for 30 min with an Akt inhibitor or left untreated, then infected for 24 h at MOI 20 with *P. gingivalis* ATCC WT, ∆PPAD, C351A PPAD, ∆FimA, ∆FimE, ∆FimC, and W83 strains. Secretion of IL-6 and IL-8 was measured by ELISA; *n* = 4−7. Results are shown as mean ± SEM; ****p < 0.0001; ***p < 0.001; **p < 0.01; *p < 0.05 compared to ATCC WT without Akt inhibitor; ns, not significant.

Next, we examined how PPAD influences *P. gingivalis*'s ability to manipulate immune and antimicrobial responses in macrophages. Crosstalk between TLR2 and C5aR receptors has been shown to activate the Pi3K/Akt pathway, which reduces phagosome maturation [3,37,38]. This pathway was also involved in inside-out signalling between TLR2 and the integrin receptor CR3, leading to increased bacterial binding [39,40]. The Akt phosphorylation status in MDMs infected with the ATCC WT and ∆PPAD strains was therefore measured at three different time points. Akt phosphorylation in MDMs was significantly higher 30 min after infection with the ATCC WT strain compared to the PPAD mutant (Figure 4A). At the same time, phosphorylation was also significantly higher in MDMs infected with the ATCC WT strain compared to the fimbriae, PPAD mutants, and W83 (Figure 4B). Fimbriae purified from the ATCC WT and ∆PPAD strains did not affect Akt phosphorylation levels, which stayed similar to untreated human MDMs (Suppl. Figure 2), suggesting that fimbriae alone might not be enough to trigger the receptor crosstalk that activates the PI3K/Akt pathway, even though citrullination was not directly observed.

The role of the PI3K/Akt pathway in the pro-inflammatory response of hMDMs infected with *P. gingivalis* strains was examined by measuring IL-6 and IL-8 production. Compared to *P. gingivalis* ATCC WT, all tested strains except for ΔFimA induced significantly lower levels of IL-6 and IL-8; although the levels induced by the FimA-deficient strain were lower than those by the parental strain, the differences were not statistically significant (Figure 4C). When hMDMs were treated with the Akt inhibitor before infection, IL-6 release was significantly reduced only after infection with the ATCC WT strain. In contrast, IL-8 secretion remained unaffected, even after infection with ATCC WT, indicating differences in how the pathways controlling IL-6 and IL-8 secretion are activated.

### Transcriptomic profiling of infected MDMs emphasises the role of accessory fimbriae subunits in *P. gingivalis* virulence

RNA-seq analysis was conducted to understand how PPAD and accessory fimbriae subunits influence the overall transcriptional profile of hMDMs. MDMs were infected with the ATCC WT strain and with isogenic strains lacking PPAD (ΔPPAD), total fimbriae (ΔFimA), and accessory subunits (∆FimC and ΔFimE). The W83 strain, which naturally lacks fimbriae [34] but produces levels of PPAD similar to the ATCC WT [11], was included for comparison. Principal component analysis (PCA) showed that mutant strains and W83 grouped between the ATCC WT and uninfected controls (Figure 5A). Additionally, heatmaps of enriched pathways demonstrated that the pathways most increased in MDMs infected with ATCC WT compared to mutant strains were related to pro-inflammatory mediators, interleukin signalling, cytokine-receptor interactions, and interferon signalling. Conversely, the most decreased pathways involved lysosomes and oxidative phosphorylation signalling (Figure 5B), supporting the idea that PPAD-mediated modification of accessory fimbriae subunits can influence phagosome maturation.

Figure 5.RNA-seq analysis of MDMs infected with various *P. gingivalis* strains. MDMs were infected for 24 h at 20 MOI with the *P. gingivalis* ATCC 33277 WT strain (ATCC WT) and its isogenic PPAD: total (∆PPAD) and fimbriae: main FimA subunit (∆FimA), accessory subunits FimE (∆FimE) and FimC (∆FimC) mutants, or W83 WT strain. A) Principal component analysis (PCA) of the entire transcriptome from MDMs infected with these *P. gingivalis* strains, with M1–M5 indicating samples from five independent donors. B) Heat maps summarising enrichment of significant signalling pathways among differentially expressed genes (DEGs) in these infected MDMs. C) DEG heatmap showing the most important DEGs from enriched signalling pathways. D) Volcano plots comparing expression patterns in MDMs infected with ATCC WT, ∆PPAD, and ∆FimA strains, as well as among PPAD, ∆FimA, ∆FimE, and ∆FimC strains. The significance (x-axis) and fold-change (y-axis) are represented as −log_10_(*p*-value) and log_2_(fold-change), respectively.

**Figure:**
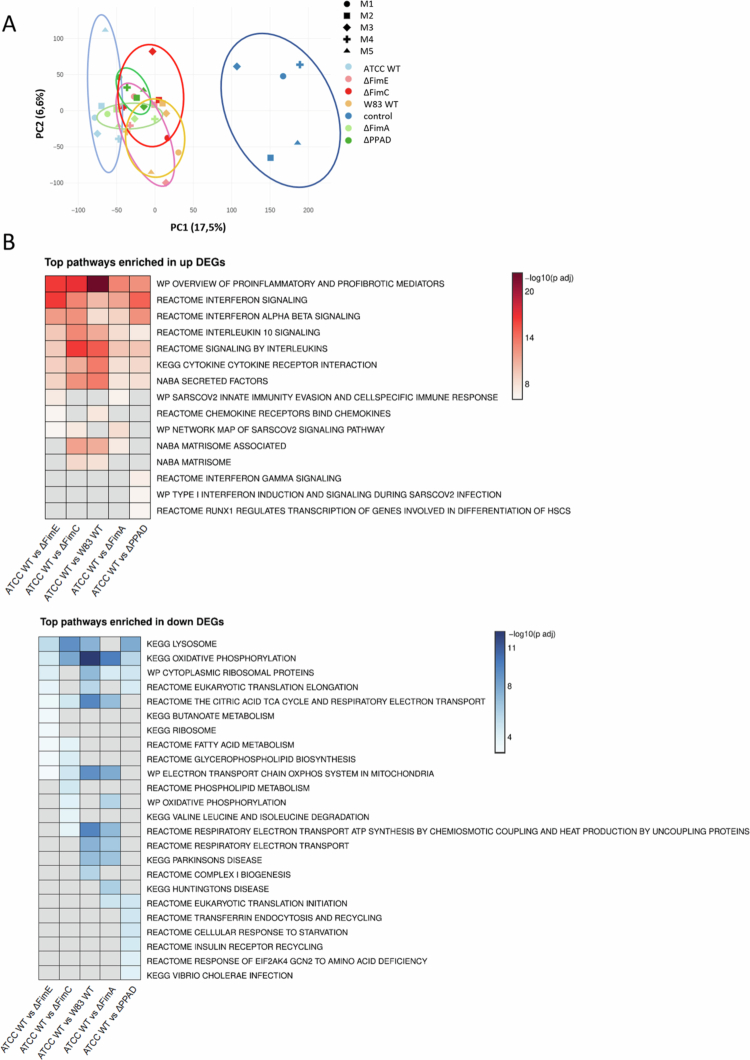

**Figure:**
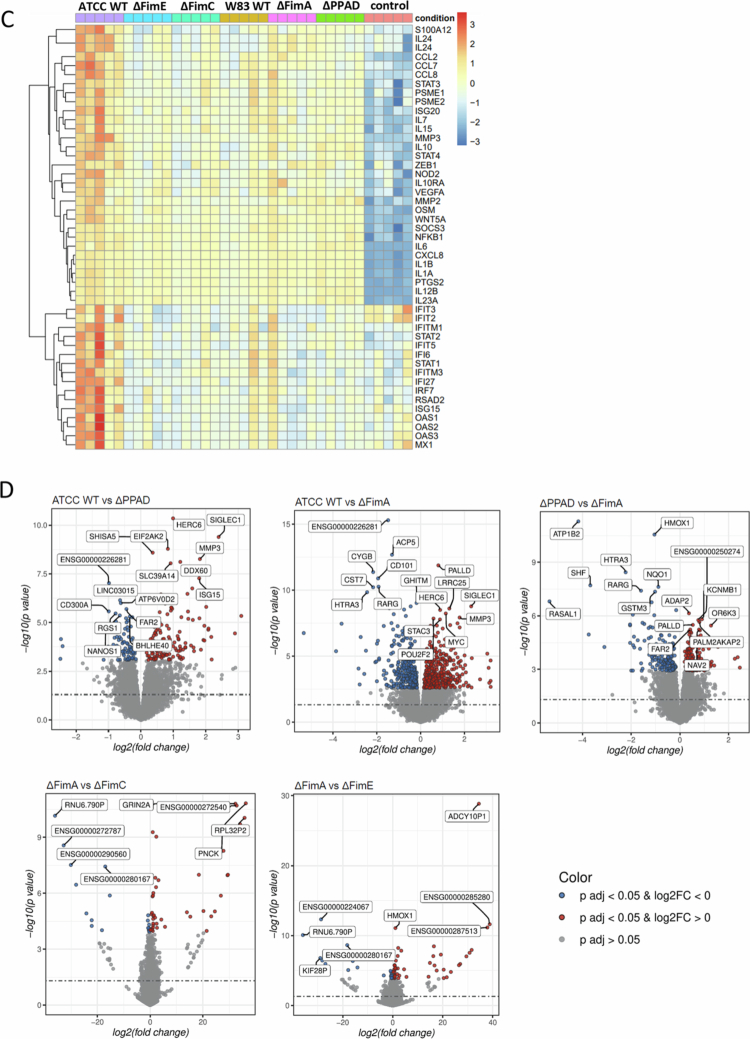

We found that the most differentially expressed genes include those linked to interferon signalling and viral infections (e.g. *IFIT, IRF, OAS, STAT* family genes, *MX1, ZEB1,* and *SOCS3*); pro-inflammatory cytokines (e.g. *IL6, IL1A, IL1B, CXCL8, CCL2, CCL7,* and *CCL8*); cytokines involved in T cell interactions (e.g. *IL10, IL2, IL7,* and *IL15*); Nod, VEGF, and Wnt signalling pathways (e.g. *NOD2, VEGFA,* and *WNT5A*); and matrix metalloproteinases (e.g. *MMP2* and *MMP3*) (Figure 5C and Suppl. Figure 3). Volcano plots showed that ΔFimA varied more from ATCC WT than ΔPPAD, while ΔFimC and ΔFimE profiles are nearly identical to ΔFimA (Suppl. Figure 4A, Figure 5D). The W83 strain exhibited the greatest variability among all comparisons (Suppl. Figure 4A, B). Overall, these findings confirm the crucial role of accessory fimbrial subunits in how *P. gingivalis* manipulates host immune responses and suggest a possible cross-talk between accessory fimbrial subunits and PPAD.

## Discussion

*P. gingivalis* has been shown to subvert host immune responses, with TLR2 playing a key role in this process. This study describes a potential mechanism by which *P. gingivalis* hijacks host defences. Although citrullination was not directly observed, the results suggest that PPAD-modified major fimbriae contribute to the pro-inflammatory responses of macrophages. Simultaneously, by altering phagocyte responses, these modified fimbriae help *P. gingivalis* survive within the bactericidal environment of phagosomes. The study demonstrated that the pro-inflammatory responses of hMDMs, indicated by the expression of *COX2* and cytokine production and secretion, depend on citrullination and the modification of accessory fimbriae subunits by PPAD.

*P. gingivalis* can evade host cells through various mechanisms involving both TLR2 and fimbriae. The so-called inside-out signalling between TLR2 and integrin CR3 (β2 integrin complement receptor 3 or CD11b/CD18) mediates the safe entry of *P. gingivalis* into macrophages [40,41]. The present study showed that total and catalytic PPAD mutants were not effectively phagocytosed by macrophages, as they either adhered to cell surfaces or remained extracellular. This may suggest that PPAD activity is important for inducing this inside-out signalling and interactions with integrins. In another mechanism, *P. gingivalis* evades killing by macrophages by inducing crosstalk between TLR2 and the complement receptor C5aR, which leads to disrupted actin polymerisation and ultimately prevents phagosome maturation [3,37]. Importantly, the Pi3K/Akt pathway is involved [40]. This study found that the levels of Akt phosphorylation were lower in macrophages infected with PPAD and fimbriae mutants compared to the WT strain, with reduced Akt phosphorylation being associated with more efficient killing of phagocytosed mutant strains. Although enhanced adhesion of PPAD mutants might be expected to facilitate invasion, our data indicate that adhesion and internalisation are uncoupled in this context, underscoring the complexity of receptor-mediated uptake and highlighting the need for future studies on phagosome maturation and trafficking. While purified fimbriae provide mechanistic insight into PPAD-dependent modulation of host signalling, infections with whole bacteria were included in our study to assess the relevance of these effects in a more physiologically representative setting.

Although we initially hypothesised that TLR2–PI3K–Akt signalling mediates the enhanced IL-6 response and intracellular survival of *P. gingivalis*, subsequent experiments showed that TLR2-blocking antibodies, TLR2 siRNA, and Akt inhibition did not significantly alter cytokine production or bacterial survival (data not shown). These inconclusive results suggest that other receptors (e.g. CR3, TLR4, or scavenger receptors), which are known to engage PI3K–Akt signalling in macrophages, might activate alternative pathways (such as CCR2-mediated PI3K–Akt activation [42], TLR4 signalling via Akt [43] or FcR-linked PI3K–Akt via SLAMF8 [44]. Our findings demonstrate that Akt activation during *P. gingivalis*–macrophage interactions is not solely triggered by TLR2 engagement, nor is it the only factor responsible for the increased IL-6 secretion caused by the ATCC 33277 strain. Instead, the data indicate complex signalling and receptor crosstalk. *P. gingivalis* interacts with multiple innate immune receptors, such as CR3, TLR4, and scavenger receptors, many of which can activate PI3K/Akt pathways in macrophages [45]. Therefore, blocking a single receptor or pathway component may not completely disrupt Akt-dependent signalling, as alternative receptors or parallel pathways could compensate. Notably, our TLR4 inhibition experiments indicate that TLR4 is unlikely to serve as a dominant alternative route to PI3K–Akt activation in response to *P. gingivalis*. Our RNA-seq data suggest engagement of a broader signalling network that may contribute to Akt modulation. In particular, genes related to Wnt signalling (such as WNT5A) were differentially regulated in macrophages infected with wild-type versus mutant strains. Wnt ligands, especially non-canonical ligands like Wnt5a, have been shown in multiple contexts to engage PI3K–Akt signalling either directly or indirectly, leading to Akt phosphorylation and downstream effects on cellular functions, including cell migration and inflammatory responses [46]. Crosstalk between the Wnt and PI3K/Akt pathways has also been documented in other systems, where PI3K/Akt activity influences *β*-catenin dynamics and Wnt target gene expression, highlighting mechanistic intersections between these pathways [47]. Although the precise role of Wnt signalling in Akt activation during *P. gingivalis*–macrophage interactions remains unclear, the co-regulation of Wnt pathway components in our transcriptomic profiles suggests that Wnt-associated signals may act in parallel with, or downstream of, classical innate receptor signalling to modulate PI3K–Akt and inflammatory outputs in this model. Overall, our results support a model where PPAD activity and fimbriae-mediated adhesion influence Akt activation and IL-6 production within a complex, interconnected signalling network. While our observations show a strong link between Akt phosphorylation and the inflammatory response induced by ATCC 33277, they do not allow us to definitively attribute these effects solely to TLR2 or Akt activity. This underscores the complex nature of host–pathogen signalling networks in macrophages and highlights the need for future studies employing genetic approaches and broader pathway analyses.

Interestingly, Akt phosphorylation is necessary to polarise macrophages into the M2 (anti-inflammatory) phenotype [48]. These M2 macrophages cannot produce ROS to eliminate bacteria and have much lower levels of iNOS than M1 macrophages [49]. Populations of M2-polarised macrophages, which are involved in tissue repair and resolving inflammation, are less common in patients with periodontitis than in healthy controls [16]. However, macrophages infected with *P. gingivalis* release large amounts of pro-inflammatory cytokines and chemokines [50], a characteristic of M1 macrophages. These findings suggest that different macrophage subtypes may be activated at various stages of disease development or that *P. gingivalis* polarises macrophages into a hybrid type, disrupting monocyte and macrophage balance and leading to dysregulated immune responses and tissue damage [51].

Analysis of the citrullinomes of the *P. gingivalis* ATCC 33277 WT and W83 strains, along with three clinical isolates, identified several citrullinated or tentatively citrullinated proteins (i.e. proteins in which arginine residues are post-translationally converted to citrulline by peptidyl arginine deiminases), including key virulence factors such as gingipains, RagA, Mfa1, and FimA [52]. However, the biological importance of these modifications was not determined. Although citrullinated FimA was detected in a clinical isolate from a patient with RA [52], our findings suggest that it is not FimA, but citrullinated accessory fimbrial proteins that act as TLR2 agonists. Direct detection of citrullination in accessory subunits was not performed, partly because of their low abundance compared to FimA and the technical difficulties in detecting citrullinated peptides [53], which may explain why citrullination of accessory fimbrial subunits was not observed in citrullinomes [52]. The potential citrullination of all or some fimbrial subunits cannot be ruled out, and thus, modification of different subunits might have distinct roles, such as TLR2 activation by accessory subunits and enhanced adhesion mediated by FimA.

RNA-seq analysis of macrophages infected with *P. gingivalis* revealed the complex interaction between the bacterium and the host immune system, especially with respect to antiviral responses and pro-inflammatory pathways. The notable increase in genes related to interferon and antiviral responses, such as *IFIT* [54], *IRF* [55], *OAS* [56], *STAT* family genes [55], *MX1* [54], *ZEB1* [57], and *SOCS3* [58], highlights the strong immune responses triggered by *P. gingivalis*, particularly through citrullinated ligands like accessory fimbriae subunits. Although *P. gingivalis* has been linked to periodontitis and viral infections, these connections are complex. For example, Herpes simplex virus was positively correlated with periodontitis [59], while *P. gingivalis* can elevate the expression of the human immunodeficiency virus coreceptor CCR5 and facilitate infection [60]. Conversely, *P. gingivalis* has been shown to inhibit SARS-CoV-2 infection [61]. Additionally, our findings align with another study indicating that, in a murine periodontitis model, interferon levels are increased, as are IFN-I levels in patients [62]. Furthermore, PPAD and fimbriae significantly contribute to the expression of cytokine genes involved in macrophage and T cell interactions, such as IL24, IL2, IL7, IL15, and IL10. This may lead to immune surveillance by suppressing T cell responses like cytotoxic activity, antigen presentation, and proliferation [63–65].

## Conclusions

We thoroughly assessed how PPAD affects inflammatory and antibacterial responses of macrophages to the fimbriated wild-type *P. gingivalis* strain ATCC 33277. The results emphasise the importance of PPAD-driven modification of fimbriae in relation to TLR2-related immune responses and *P. gingivalis* virulence.

## Supplementary Material

MDMs_FimCDE_supplementary_materials.docx MDMs_FimCDE_supplementary_materials.docx

## Data Availability

The RNA-seq data generated in this study have been deposited in the NCBI Gene Expression Omnibus (GEO) under accession number GSE304218. The data can be accessed at the following link: https://www.ncbi.nlm.nih.gov/geo/query/acc.cgi?acc=GSE304218 using the access token: ipwbeyugzlgnzyd. All other data supporting the findings of this study are available from the corresponding author upon reasonable request.

## References

[1] Hajishengallis G. Periodontitis: from microbial immune subversion to systemic inflammation. Nat Rev Immunol. 2015;15(1):30–44. doi: 10.1038/nri3785 25534621 PMC4276050

[2] Hajishengallis G, Darveau RP, Curtis MA. The keystone-pathogen hypothesis. Nat Rev Microbiol. 2012;10(10):717–725. doi: 10.1038/nrmicro2873 22941505 PMC3498498

[3] Jia L, Han N, Du J, et al. Pathogenesis of important virulence factors of *porphyromonas gingivalis* via toll-like receptors. Front Cell Infect Microbiol. 2019;9(July):262. doi: 10.3389/fcimb.2019.00262 31380305 PMC6657652

[4] Veith PD, Chen YY, Gorasia DG, et al. *Porphyromonas gingivalis* outer membrane vesicles exclusively contain outer membrane and periplasmic proteins and carry a cargo enriched with virulence factors. J Proteome Res. 2014;13(5):2420–2432. doi: 10.1021/pr401227e 24620993

[5] Gabarrini G, Chlebowicz MA, Vega Quiroz ME, et al. Conserved citrullinating exoenzymes in porphyromonas species. J Dent Res. 2018;97(5):556–562. doi: 10.1177/0022034517747575 29298553

[6] Goulas T, Mizgalska D, Garcia-Ferrer I, et al. Structure and mechanism of a bacterial host-protein citrullinating virulence factor, *porphyromonas gingivalis* peptidylarginine deiminase. Sci Rep. 2015;5(April):11969. doi: 10.1038/srep11969 26132828 PMC4487231

[7] Lagosz-Cwik KB, Wielento A, Lipska W, et al. hTERT-immortalized gingival fibroblasts respond to cytokines but fail to mimic primary cell responses to *porphyromonas gingivalis*. Sci Rep. 2021;11(1):10770. doi: 10.1038/s41598-021-90037-5 34031466 PMC8144196

[8] Wielento A, Lagosz-Cwik KB, Potempa J, et al. The role of gingival fibroblasts in the pathogenesis of periodontitis. J Dent Res. 2023;102(5):489–496. doi: 10.1177/00220345231151921 36883660 PMC10249005

[9] Hajishengallis G, Tapping RI, Harokopakis E, et al. Differential interactions of fimbriae and lipopolysaccharide from *porphyromonas gingivalis* with the toll-like receptor 2-centred pattern recognition apparatus. Cell Microbiol. 2006;8(10):1557–1570. doi: 10.1111/j.1462-5822.2006.00730.x 16984411

[10] Hasegawa Y, Nagano K. *Porphyromonas gingivalis* FimA and Mfa1 fimbriae: current insights on localization, function, biogenesis, and genotype. Jpn Dent Sci Rev. 2021;57:190–200. doi: 10.1016/j.jdsr.2021.09.003 34691295 PMC8512630

[11] Wielento A, Bereta GP, Łagosz-Ćwik KB, et al. TLR2 activation by *porphyromonas gingivalis* requires both PPAD activity and fimbriae. Front Immunol. 2022;13(April):823685. doi: 10.3389/fimmu.2022.823685 35432342 PMC9010743

[12] Wielento A, Bereta GP, Szczęśniak K, et al. Accessory fimbrial subunits and PPAD are necessary for TLR2 activation by *porphyromonas gingivalis*. Mol Oral Microbiol. 2023;38(4):334–346. doi: 10.1111/omi.12427 37347653

[13] Wielento A, Bereta GP, Łagosz-Ćwik KB, et al. TLR2 activation by *porphyromonas gingivalis* requires both PPAD activity and fimbriae. Front Immunol. 2022;13(April):823685. doi: 10.3389/fimmu.2022.823685 35432342 PMC9010743

[14] Wielento A, Bereta GP, Szczęśniak K, et al. Accessory fimbrial subunits and PPAD are necessary for TLR2 activation by *porphyromonas gingivalis*. Mol Oral Microbiol. 2023;38(4):334–346. doi: 10.1111/omi.12427 37347653

[15] Sun X, Gao J, Meng X, et al. Polarized macrophages in periodontitis: characteristics, function, and molecular signaling. Front Immunol. 2021;12(December):1–18. doi: 10.3389/fimmu.2021.763334 PMC8688840 34950140

[16] Lin J, Huang D, Xu H, et al. Macrophages: a communication network linking *porphyromonas gingivalis* infection and associated systemic diseases. Front Immunol. 2022;13(July):1–11. doi: 10.3389/fimmu.2022.952040 PMC9363567 35967399

[17] Wang M, Krauss JL, Domon H, et al. Microbial hijacking of complement-toll-like receptor crosstalk. Sci Signal. 2010;3(109):ra11. doi: 10.1126/scisignal.2000697 20159852 PMC2824906

[18] Gawron K, Bereta G, Nowakowska Z, et al. Peptidylarginine deiminase from *porphyromonas gingivalis* contributes to infection of gingival fibroblasts and induction of prostaglandin E 2 -signaling pathway. Mol Oral Microbiol. 2014;29(6):321–332. doi: 10.1111/omi.12081 25176110 PMC4617314

[19] Love RM, McMillan MD, Park Y, et al. Coinvasion of dentinal tubules by *porphyromonas gingivalis* and streptococcus gordonii depends upon binding specificity of streptococcal antigen I/II adhesin. Infect Immun. 2000;68(3):1359–1365. doi: 10.1128/IAI.68.3.1359-1365.2000 10678948 PMC97289

[20] Hongo H, Osano E, Ozeki M, et al. Characterization of an outer membrane protein gene, pgmA, and its gene product from *porphyromonas gingivalis*. Microbiol Immunol. 1999;43(10):937–946. doi: 10.1111/j.1348-0421.1999.tb03354.x 10585140

[21] Nishiyama SI, Murakami Y, Nagata H, et al. Involvement of minor components associated with the FimA fimbriae of *porphyromonas gingivali**s* in adhesive functions. Microbiology. 2007;153(Pt 6):1916–1925. doi: 10.1099/mic.0.2006/005561-0 17526848

[22] Kelly A, Grabiec AM, Travis MA. Culture of human monocyte-derived macrophages. In Methods in molecular biology (Vol. 1784). Clifton, N.J.: Springer; 2018. p. 1–11 ). doi: 10.1007/978-1-4939-7837-3_1 29761383

[23] Dobin A, Davis CA, Schlesinger F, et al. STAR: ultrafast universal RNA-seq aligner. Bioinformatics. 2013;29(1):15–21. doi: 10.1093/bioinformatics/bts635 23104886 PMC3530905

[24] Liao Y, Smyth GK, Shi W. featureCounts: an efficient general purpose program for assigning sequence reads to genomic features. Bioinformatics. 2014;30(7):923–930. doi: 10.1093/bioinformatics/btt656 24227677

[25] Leek JT, Johnson WE, Parker HS, et al. The sva package for removing batch effects and other unwanted variation in high-throughput experiments. Bioinformatics. 2012;28(6):882–883. doi: 10.1093/bioinformatics/bts034 22257669 PMC3307112

[26] Love MI, Huber W, Anders S. Moderated estimation of fold change and dispersion for RNA-seq data with DESeq2. Genome Biol. 2014;15(12):550. doi: 10.1186/s13059-014-0550-8 25516281 PMC4302049

[27] Korotkevich G, Sukhov V, Budin N, et al. Fast gene set enrichment analysis. bioRxiv. 2021;60012. doi: 10.1101/060012

[28] Liberzon A, Subramanian A, Pinchback R, et al. Molecular signatures database (MSigDB) 3.0. Bioinformatics. 2011;27(12):1739–1740. doi: 10.1093/bioinformatics/btr260 21546393 PMC3106198

[29] Wickham H. Ggplot2: Elegant Graphics for Data Analysis. Springer-Verlag New York; 2016. p. 189–201. doi: 10.1007/978-3-319-24277-4_9

[30] Kolde R. 2019. Pretty Heatmaps (RRID: SCR_016418). Published online. https://cran.r-project.org/web/packages/pheatmap/pheatmap.pdf

[31] Nishiyama SI, Murakami Y, Nagata H, et al. Involvement of minor components associated with the FimA fimbriae of *porphyromonas gingivalis* in adhesive functions. Microbiology (N Y). 2007;153(6):1916–1925. doi: 10.1099/mic.0.2006/005561-0 17526848

[32] Lenzo JC, O'Brien-Simpson NM, Cecil J, et al. Determination of active phagocytosis of unopsonized *porphyromonas gingivalis* by macrophages and neutrophils using the ph-sensitive fluorescent dye pHrodo. Infect Immun. 2016;84(6):1753–1760. doi: 10.1128/IAI.01482-15 27021243 PMC4907136

[33] Love RM, McMillan MD, Park Y, et al. Coinvasion of dentinal tubules by *porphyromonas gingivalis* and streptococcus gordonii depends upon binding specificity of streptococcal antigen I/II adhesin. Infect Immun. 2000;68(3):1359–1365. doi: 10.1128/IAI.68.3.1359-1365.2000 10678948 PMC97289

[34] Nelson KE, Fleischmann RD, DeBoy RT, et al. Complete genome sequence of the oral pathogenic bacterium *porphyromonas gingivalis* strain W83. J Bacteriol. 2003;185(18):5591–5601. doi: 10.1128/JB.185.18.5591-5601.2003 12949112 PMC193775

[35] Wang M, Shakhatreh MAK, James D, et al. Fimbrial proteins of *porphyromonas gingivalis* mediate in vivo virulence and exploit TLR2 and complement receptor 3 to persist in macrophages. J Immunol. 2007;179(4):2349–2358. doi: 10.4049/jimmunol.179.4.2349 17675496

[36] Vermilyea DM, Ottenberg GK, Davey ME. Citrullination mediated by PPAD constrains biofilm formation in *P. Gingivalis* strain 381. NPJ Biofilms Microbiomes. 2019;5(1):1–11. doi: 10.1038/s41522-019-0081-x 32029738 PMC6367333

[37] Maekawa T, Krauss JL, Abe T, et al. *Porphyromonas gingivalis* manipulates complement and TLR signaling to uncouple bacterial clearance from inflammation and promote dysbiosis. Cell Host Microbe. 2014;15(6):768–778. doi: 10.1016/j.chom.2014.05.012 24922578 PMC4071223

[38] Makkawi H, Hoch S, Burns E, et al. *Porphyromonas gingivalis* stimulates TLR2-PI3K signaling to escape immune clearance and induce bone resorption independently of MyD88. Front Cell Infect Microbiol. 2017;7(AUG):359. doi: 10.3389/fcimb.2017.00359 28848717 PMC5550410

[39] Harokopakis E, Hajishengallis G. Integrin activation by bacterial fimbriae through a pathway involving CD14, toll-like receptor 2, and phosphatidylinositol-3-kinase. Eur J Immunol. 2005;35(4):1201–1210. doi: 10.1002/eji.200425883 15739163

[40] Hajishengallis G, Wang M, Liang S. Induction of distinct TLR2-Mediated proinflammatory and proadhesive signaling pathways in response to *porphyromonas gingivalis* fimbriae. J Immunol. 2009;182(11):6690–6696. doi: 10.4049/jimmunol.0900524 19454663 PMC2685460

[41] Harokopakis E, Hajishengallis G. Integrin activation by bacterial fimbriae through a pathway involving CD14, tool-like receptor 2, and phosphatidylinositol-3-kinase. Eur J Immunol. 2005;35(4):1201–1210. doi: 10.1002/eji.200425883 15739163

[42] Dong Y, Dong Y, Zhu C, et al. Targeting CCL2-CCR2 signaling pathway alleviates macrophage dysfunction in COPD via PI3K-AKT axis. Cell Commun Signal. 2024;22(1):364. doi: 10.1186/s12964-024-01746-z 39014433 PMC11253350

[43] Bauerfeld CP, Rastogi R, Pirockinaite G, et al. TLR4-mediated AKT activation is MyD88/TRIF dependent and critical for induction of oxidative phosphorylation and mitochondrial transcription factor A in murine macrophages. J Immunol. 2012;188(6):2847–2857. doi: 10.4049/jimmunol.1102157 22312125 PMC3294201

[44] Liu Z, Hu J, Han X, et al. SLAMF8 regulates fc receptor-mediated phagocytosis in mouse macrophage cells through PI3K-Akt signaling. Immunol Lett. 2025;273:106990. doi: 10.1016/j.imlet.2025.106990 39983459

[45] Hajishengallis G. Immune evasion strategies of *porphyromonas gingivalis*. J Oral Biosci. 2011;53(3):233–240. doi: 10.2330/joralbiosci.53.233 22162663 PMC3231999

[46] Zhang A, He S, Sun X, et al. Wnt5a promotes migration of human osteosarcoma cells by triggering a phosphatidylinositol-3 kinase/Akt signals. Cancer Cell Int. 2014;14(1):15. doi: 10.1186/1475-2867-14-15 24524196 PMC3976035

[47] Fleming-de-Moraes CD, Rocha MR, Tessmann JW, et al. Crosstalk between PI3K/Akt and Wnt/β-catenin pathways promote colorectal cancer progression regardless of mutational status. Cancer Biol Ther. 2022;23(1):1–13. doi: 10.1080/15384047.2022.2108690 PMC9367664 35944058

[48] Vergadi E, Ieronymaki E, Lyroni K, et al. Akt signaling pathway in macrophage activation and M1/M2 polarization. J Immunol. 2017;198(3):1006–1014. doi: 10.4049/jimmunol.1601515 28115590

[49] Lam RS, O'Brien-Simpson NM, Holden JA, et al. Unprimed, M1 and M2 macrophages differentially interact with *porphyromonas gingivalis*. PLoS One. 2016;11(7):1–17. doi: 10.1371/journal.pone.0158629 PMC4934774 27383471

[50] Hou Y, Yu H, Liu X, et al. Gingipain of *porphyromonas gingivalis* manipulates M1 macrophage polarization through C5a pathway. In Vitro Cell Dev Biol Anim. 2017;53(7):593–603. doi: 10.1007/s11626-017-0164-z 28634882

[51] Wang W, Zheng C, Yang J, et al. Intersection between macrophages and periodontal pathogens in periodontitis. J Leukoc Biol. 2021;110(3):577–583. doi: 10.1002/JLB.4MR0421-756R 34028883

[52] Stobernack T, Glasner C, Junker S, et al. Extracellular proteome and citrullinome of the oral pathogen *porphyromonas gingivalis*. J Proteome Res. 2016;15(12):4532–4543. doi: 10.1021/acs.jproteome.6b00634 27712078

[53] Verheul MK, van Veelen PA, van Delft MAM, et al. Pitfalls in the detection of citrullination and carbamylation. Autoimmun Rev. 2018;17(2):136–141. doi: 10.1016/j.autrev.2017.11.017 29203292

[54] Tirumurugaan K, Pawar R, Dhinakar Raj G, et al. RNAseq reveals the contribution of interferon stimulated genes to the increased host defense and decreased PPR viral replication in cattle. Viruses. 2020;12(4):463. doi: 10.3390/v12040463 32325933 PMC7232496

[55] Wang J, Li H, Xue B, et al. IRF1 promotes the innate immune response to viral infection by enhancing the activation of IRF3. J Virol. 2020;94(22). doi: 10.1128/JVI.01231-20 PMC7592201 32878885

[56] Eckhart L, Sipos W. Differential loss of OAS genes indicates diversification of antiviral immunity in mammals. Vaccines (Basel). 2023;11(2):419. doi: 10.3390/vaccines11020419 36851296 PMC9964502

[57] Yu X, Wang Z, Mertz JE. ZEB1 regulates the latent-lytic switch in infection by epstein-barr virus. PLoS Pathog. 2007;3(12):e194. doi: 10.1371/journal.ppat.0030194 18085824 PMC2134958

[58] Yan D, Li G, Yuan Y, et al. SOCS3 inhibiting JAK-STAT pathway enhances oncolytic adenovirus efficacy by potentiating viral replication and T-cell activation. Cancer Gene Ther. 2024;31(3):397–409. doi: 10.1038/s41417-023-00710-2 38102464

[59] Song Y, Liu N, Gao L, et al. Association between human herpes simplex virus and periodontitis: results from the continuous national health and nutrition examination survey 2009-2014. BMC Oral Health. 2023;23(1):675. doi: 10.1186/s12903-023-03416-x 37723536 PMC10507957

[60] Giacaman RA, Asrani AC, Gebhard KH, et al. *Porphyromonas gingivalis* induces CCR5-dependent transfer of infectious HIV-1 from oral keratinocytes to permissive cells. Retrovirology. 2008;5:29. doi: 10.1186/1742-4690-5-29 18371227 PMC2292744

[61] Bontempo A, Chirino A, Heidari A, et al. Inhibition of SARS-CoV-2 infection by *porphyromonas gingivalis* and the oral microbiome. Microbiol Spectr. 2024;10(12):e0059924. doi: 10.1128/spectrum.00599-24 PMC11448423 39162507

[62] Mizraji G, Nassar M, Segev H, et al. *Porphyromonas gingivalis* promotes unrestrained type I interferon production by dysregulating TAM signaling via MYD88 degradation. Cell Rep. 2017;18(2):419–431. doi: 10.1016/j.celrep.2016.12.047 28076786

[63] Sun L, Girnary M, Wang L, et al. IL-10 dampens an IL-17-Mediated periodontitis-associated inflammatory network. J Immunol. 2020;204(8):2177–2191. doi: 10.4049/jimmunol.1900532 32169848 PMC7840149

[64] Zhong Y, Zhang X, Chong W. Interleukin-24 immunobiology and its roles in inflammatory diseases. Int J Mol Sci. 2022;23(2):627. doi: 10.3390/ijms23020627 35054813 PMC8776082

[65] ElKassar N, Gress RE. An overview of IL-7 biology and its use in immunotherapy. J Immunotoxicol. 2010;7(1):1–7. doi: 10.3109/15476910903453296 20017587 PMC2826542

