# Supplementary material for: Macrophage activation and invasion by P. gingivalis is modulated by PPAD and accessory fimbriae subunits
Source: J Oral Microbiol. 2026 Mar 3;18(1):2638646. doi: 10.1080/20002297.2026.2638646 (PMC12958381; doi:10.1080/20002297.2026.2638646)
Supplement: MDMs_FimCDE_supplementary_materials.docx [file ZJOM_A_2638646_SM3647.docx]

**Supplementary figures**


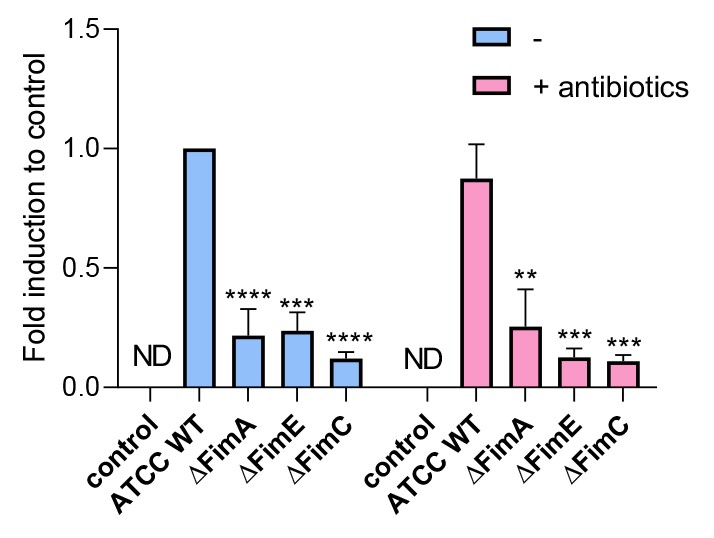


**Suppl. Fig. 1. Accessory fimbrial subunits are essential for *P. gingivalis* resistance to killing by macrophages.** MDMs were infected for 30 min at 20 MOI with *P. gingivalis* ATCC WT, ∆FimA, ∆FimE and ∆FimC strains and *P. gingivalis* intracellular survival was determined by antibiotic protection assays. After 5 days in anaerobic culture, the numbers of colony forming units (CFU) were calculated; n=3. Results are presented as mean +/- SEM. **** p<0.0001; *** p<0.001; ** p<0.01 compared with ATCC WT strain; ND, not detected.


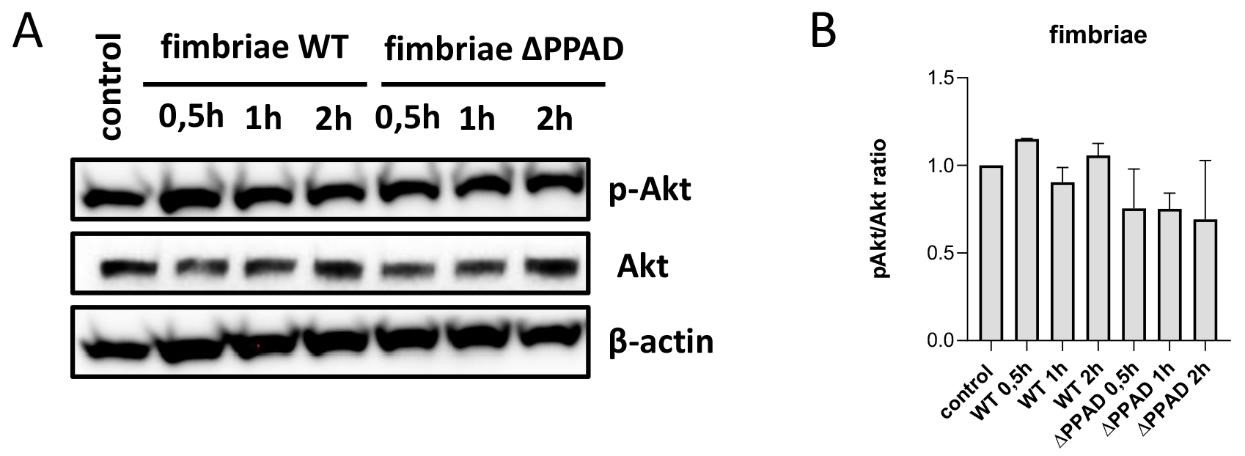


**Suppl. Fig. 2. Fimbriae alone fail to activate AKT phosphorylation.** MDMs were treated for 30 min, 1 h or 2 h with purified fimbriae (1 μg/ml) from *P. gingivalis* WT and ∆PPAD strains, and the levels of Akt phosphorylation were determined by western blot analysis, with total Akt as the control and β-actin as the loading control. **A)** Representative blots are shown and **B)** the results of densitometry (n=3) are presented as the mean ± SEM.


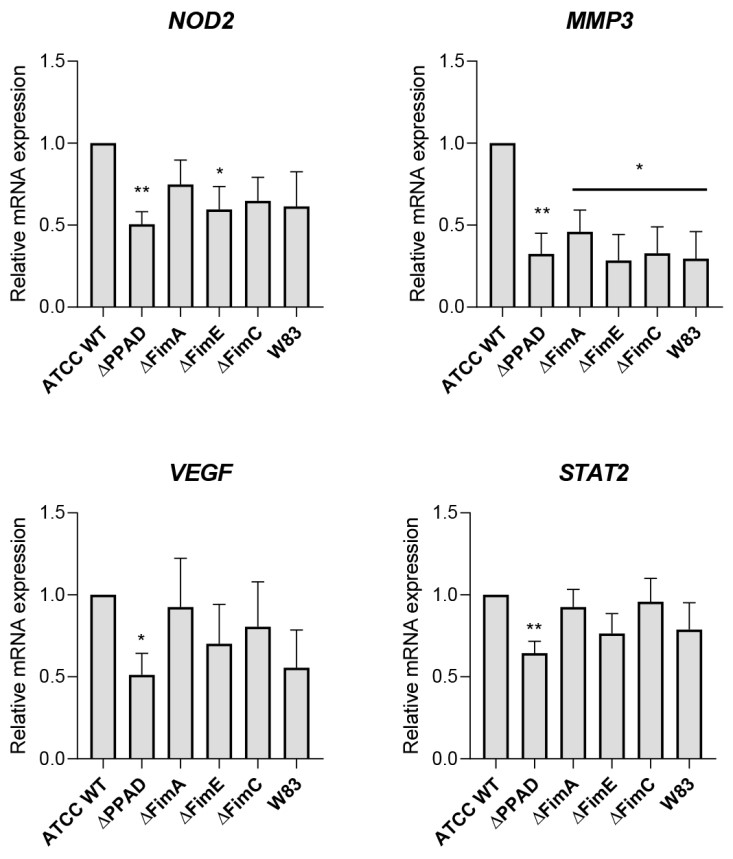


**Suppl. Fig. 3. Validation of the RNA-seq data of MDMs infected with various *P. gingivalis* strains.** Relative mRNA expression of the chosen genes (*NOD2*, *MMP3*, *VEGF*, *STAT2*) differentially expressed from enriched signaling pathways. Data represent the mean ± SEM compared with ATCC WT strain , ** p<0.01; * p<0.05.


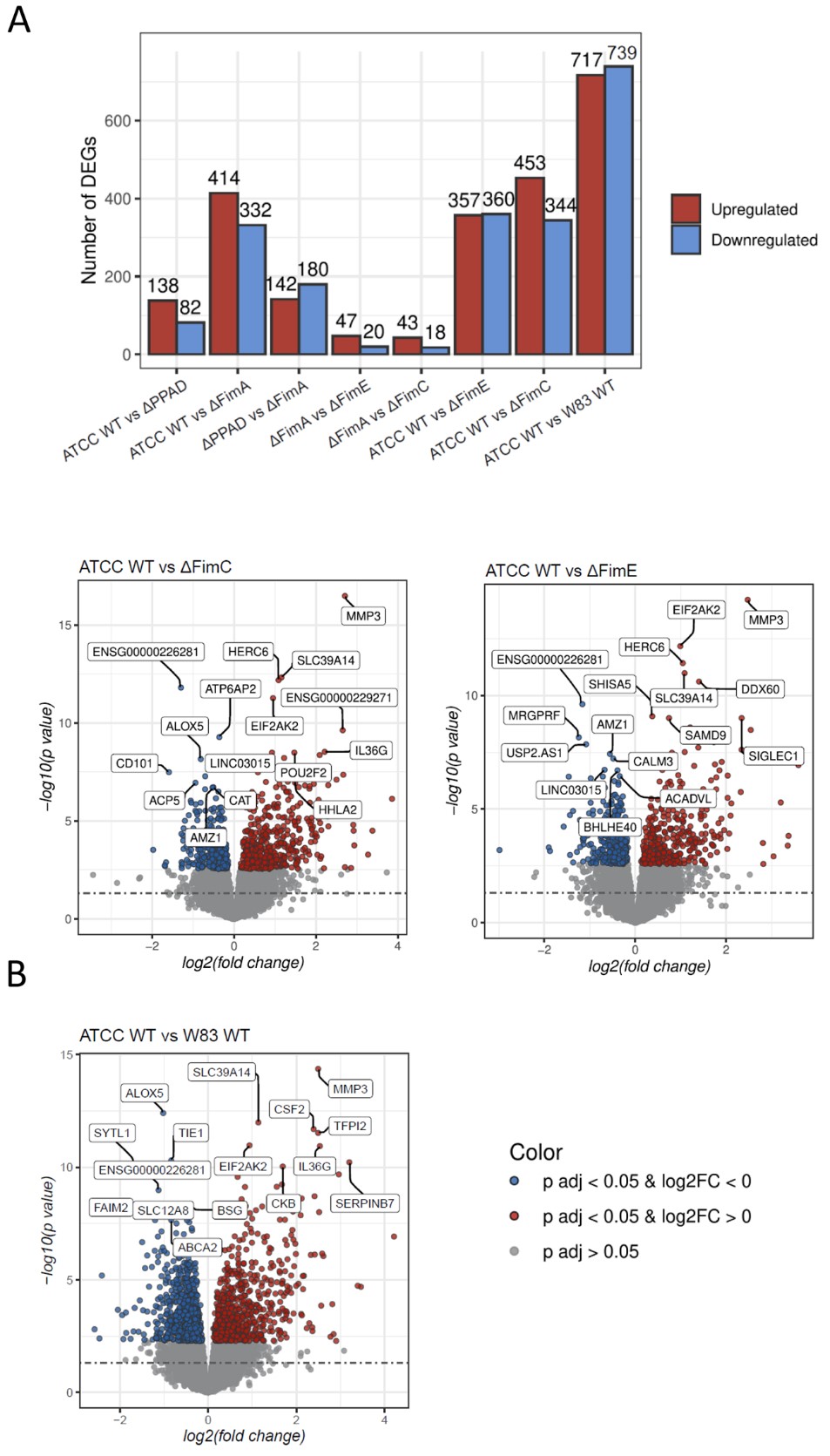


**Suppl. Fig. 4. Volcano plots comparing expression patterns in MDMs infected with *P. gingivalis* ATCC 33277 WT (ATCC WT) and accessory fimbriae subunits mutants (∆FimE or ∆FimC) or W83 WT strain.**  The significance (x-axis) and fold-change (y-axis) were converted to −log_10_(p-value) and log_2_(fold-change), respectively.
